# Isolated superior mesenteric artery traumatic injury following body height fall across a log

**DOI:** 10.1093/jscr/rjac500

**Published:** 2022-11-14

**Authors:** Munasinghe Silva, Aathavan Shanmuga Anandan

**Affiliations:** Faculty of Medicine, The University of Queensland, Brisbane, Queensland, Australia; Faculty of Medicine, The University of Queensland, Brisbane, Queensland, Australia

## Abstract

This case outlines a 71-year-old male falling from body height horizontally across a log with isolated superior mesenteric artery vascular injury, a rarity in minor blunt force trauma. A computed tomography abdomen and pelvis with contrast was ordered revealing active contrast blush arising from a major mesenteric vessel consistent with active arterial bleeding. Bowels and solid intra-abdominal organs were spared from any injuries. He underwent an emergency laparotomy following immediate resuscitation. The bleeding was controlled with clamping of the proximal and distal segments of the superior mesenteric artery (SMA). A severed branch of SMA was identified and ligated to stop the haemorrhage. The patient’s recovery was uncomplicated, and he was discharged home on post-operative day 8. This case report highlights a unique mechanism of injury causing a rare injury pattern, emphasizing the importance of early recognition and definitive surgical intervention.

## INTRODUCTION

Trauma is a growing burden of disease, accounting for 13% of disability-adjusted life years and 11% of global mortality rate [1]. The most common trauma presentation to emergency department (ED), in adults and children alike, is abdominal trauma with solid organ injuries caused by blunt force [2]. Damages due to penetrating trauma are isolated and more reliably diagnosable due to easier localization of injuries [3]. Comparatively, blunt injuries result in diffuse injury patterns with subtle clinical signs, resulting in delayed intervention and poorer management [3]. Visceral artery injuries from blunt trauma can lead to devastating outcomes and prompt diagnosis and urgent surgical or interventional radiology management are required to achieve the best morbidity and mortality outcomes. General management for both blunt and penetrative trauma warrants resuscitation followed by definitive surgical or interventional radiology treatment as required. In this case, we present a case characterized by a severe blunt force injury causing highly focalized vascular injury threatening the life.

## CASE REPORT

This case report outlines the presentation of a 71-year-old male who presented to a regional hospital following a horizontal fall from body height onto a log while cutting a banana tree resulting in isolated superior mesenteric artery (SMA) vascular injury.

### Presentation and Patient Background

A 71-year-old male presented to a regional hospital following a horizontal fall from body height onto a log while cutting a banana tree. The patient arrived at the emergency department (ED) complaining of severe abdominal pain, and nausea but denied vomiting or per rectal (PR) bleeding. He described no chest pain or presyncope preceding the fall.

His medical history was significant for asthma, chronic obstructive pulmonary disease, and B-cell lymphoma. His surgical history included laparotomy for a small bowel obstruction and a small bowel resection for the lymphoma. He was a heavy smoker with social alcohol consumption. His current medication list included fish oil and salbutamol.

On examination, the patient was conscious, although pale and diaphoretic, hypotensive and tachycardic. He had an acute abdomen with four-quadrant peritonism. He had no other injuries on physical exam and no signs of head trauma. There were no signs of bruising on the abdomen or the chest wall. He was resuscitated according to the trauma resuscitation protocol and the massive transfusion protocol was initiated in ED. Intravenous tranexamic acid was started to minimize blood loss. Prophylactic triple therapy antibiotics of metronidazole, gentamycin and ampicillin were commenced. While in the ED, his haemoglobin dropped rapidly, and he became acidotic and uremic. A computed tomography (CT) trauma series with contrast was organized and the arterial phase study showed an active contrast blush arising from a mesenteric vessel consistent with active arterial bleeding.

A large hyperdense collection suggestive of a haematoma was seen within the mid to upper abdomen measuring 95 × 37 × 48 mm. There was a small collection of blood within the pelvis. There was no evidence to suggest an active gastrointestinal bleed. No evidence of a hepatic or splenic laceration was identified. The kidneys were intact. The adrenal glands and pancreas were unremarkable. No fractures anywhere in the body. Chest X-ray was unremarkable.

The patient underwent emergency laparotomy due to haemodynamically instability.

A large intra-abdominal haematoma with active bleeding was noted. The haematoma was evacuated and the superior mesenteric artery (SMA) and inferior mesenteric artery (IMA) were clamped to control the active bleeding. A severed SMA branch was identified under a haematoma at the root of the mesentery and ligated with polypropylene stitch for definitive management of the haemorrhage. Floseal was syringed into the mesenteric bed. The rest of the peritoneal survey showed no abnormalities detected with the bowels viable at the end of the case. The patient was transferred to the intensive care unit (ICU) and extubated the following day. He was transferred to the medical ward on the second day, and he was discharged home after an uncomplicated recovery period.

## DISCUSSION

Superior mesenteric artery (SMA) injury is a rare and lethal pathology, generally comprising <1% of cases at trauma centres [4]. Yet, the true incidence is suspected to be greater given these cases tend to be lethal due to extensive exsanguination causing early deaths prior to trauma centre presentation. Secondary mortality is due to sepsis, multiorgan failure and secondary to ischemic bowel following visceral arterial injury [5]. No definitive guidelines exist regarding the approach to the management of SMA injuries [6]. A key feature of this case report’s rarity is associated with the mechanism of injury based on two factors: (i) penetrating trauma is more commonly associated with vascular injury and (ii) blunt injuries present with a diffuse injury pattern. Penetrating injuries such as gunshot and stab wounds tend to represent ~ 80–95% of visceral vascular injuries [7]. The remainder of 5–20% of visceral vascular trauma is associated with motor vehicle collisions and falls [5, 8]. Two primary pathophysiological theories have been postulated as the cause of blunt visceral vascular injury: (a) arterial avulsion secondary to shearing forces between the junction of unfixed bowel and the fixed retroperitoneal portion, and (b) arterial compression between the lumbar spine and the blunt vector [9, 10]. Although the blunt mechanism of injury may have been misleading, the typical acute abdomen presentation with four quadrant peritonism and signs of shock was consistent with the diagnosis. Although dependent on the force of injury, blunt trauma has a greater association with diffuse injury patterns. The absence of any injury external to the SMA injury in this case report highlights an unexpected injury pattern. SMA injuries can occur at any segment of its course. Fullen *et al*. in 1972 described an injury classification based on the segment of the SMA and the severity of ischemia [11]. There are four major zones of SMA according to the classification (Table 1).

**Table 1:** Fullen’s anatomic classification of superior mesenteric artery injury by zone and by grade

Zone	Segment of superior mesenteric artery	Grade	Category	Bowel segments affected
I	Trunk proximal to first major branch (pancreaticoduodenal)	I	Maximal	Jejunum, ileum, right colon
II	Trunk between inferior pancreaticoduodenal and middle colic	II	Moderate	Major segment, small bowel and/or right colon
III	Trunk distal to middle colic	II	Minimal	Minor segment or segments, small bowel or right colon
IV	Segmental branches, jejunal, ileal or colic	IV	None	No ischemic bowel

Computed tomography (CT) of the abdomen and pelvis remains the mainstay investigation for diagnosis of mesenteric arterial injury [12]. Following confirmatory imaging, an exploratory laparotomy is recommended to assess for further injury and commence surgical intervention [6]. Numerous surgical treatment options exist for SMA injury.

Immediate achievement of proximal and distal control of the vessel is the key to manage any vascular injury successfully. Due to its protected location and the complex lymphatic channels and celiac plexus, the exposure of the proximal SMA injury can be extremely difficult [13]. The most employed strategy is vessel ligation (51%), followed by primary repair (40%) and interposition graft (6%) [4]. In this case report, arterial ligation and Floseal injection, a haemostatic agent, were the primary interventions to minimize exsanguination. The primary risk associated with vessel ligation, particularly in the SMA, is significant bowel ischemia [5]. Bowel areas extending from the ligament of Treitz to the ascending colon are prone to necrosis [13]. It can be quite challenging for regional hospital surgeons as there is no proper guidelines for SMA injury management and the lack of training in vascular injury management with the general surgical training program in Australia [6]. As this patient presented haemodynamically unstable to a regional hospital, endovascular therapy was not a viable treatment option, as these require tertiary care.

Overall, this case report outlines a 71-year-old male who presented to a regional hospital following a horizontal fall from body height across a log while cutting a banana tree resulting in isolated superior mesenteric artery injury. Early stabilization, intervention and surgical management resulted in good post-operative recovery with nil complications at follow-up. The unique mechanism of injury and injury pattern sustained highlights the importance of consideration of rare injuries in atypical presentations.
